# Determination of FAT-1 desaturase activity and substrate preference

**DOI:** 10.1016/j.jlr.2025.100945

**Published:** 2025-11-14

**Authors:** Xiumei Xu, Yanli Wang, Yao Xu, Yanan Yao, Lin Fu, Shenglin He, Jing Leng, Qun Lu, Xiaoju Zou, Bin Liang

**Affiliations:** 1 Center for Life Sciences, Yunnan Key Laboratory of Cell Metabolism and Diseases, School of Life Sciences, Yunnan University, Kunming, China; 3 Southwest United Graduate School, Kunming, China; 2 College of Chinese Materia Medica and Yunnan Key Laboratory of Southern Medicinal Utilization, Yunnan University of Chinese Medicine, Kunming, Yunnan, China

**Keywords:** *Caenorhabditis elegans*, FAT-1 (Δ15-desaturase), PUFAs, C20:4n6 (arachidonic acid), C20:5n3 (EPA)

## Abstract

n-3 PUFAs possess numerous health benefits. The FAT-1 desaturase in the model organism *Caenorhabditis elegans* is a Δ15-desaturase that converts n-6 PUFAs into n-3 PUFAs. Transgenic expression of FAT-1 has been used in organisms, such as pigs, mice, and fish, to improve n-3 PUFA levels. However, the determination of FAT-1 activity and substrate preference per se remains unclear. AlphaFold structure prediction revealed that FAT-1 is an integral membrane protein located in the endoplasmic reticulum, and it consists of four transmembrane helices (1-4) with a functional CYTB5 domain in the N terminus and a desaturase domain containing three histidine-rich sequences (His boxes) in the C terminus. A small region in the desaturase domain containing amino acids 210–217, especially G212, G216, and S217, is essential for its activity. FAT-1 can convert all four n-6 PUFAs to corresponding or downstream n-3 PUFAs in both *C. elegans* and mammalian cells and may prioritize the conversion of C20:4n6 (arachidonic acid) to C20:5n3 (EPA). These results uncover the significant mechanism of the activity and substrate preference of the FAT-1 desaturase, providing insights into the transgenic application of FAT-1.

n-3 PUFAs, such as alpha-linolenic acid (C18:3n3), EPA (C20:5n3), and DHA (C22:6n3), are involved in various physiological functions in human health, including anti-inflammatory effects, lipid regulation, promotion of brain development, protection of cardiovascular health, and so on. Metabolic disorders of n-3 PUFAs are associated with numerous human diseases, such as inflammation and autoimmune diseases (1, 2), cardiovascular diseases (3, 4), cancer (5, 6, 7), neurodegenerative diseases (8, 9, 10), metabolic disorders (11, 12), as well as others. For example, EPA and DHA are essential components of cell membranes, regulating cellular signal transduction and gene expression, and they also exhibit anti-inflammatory and antithrombotic effects, which are helpful in preventing cardiovascular diseases (13). DHA is crucial for brain and retinal development, with particularly high demand during pregnancy and infancy (14). In addition, n-3 PUFAs have been found to potentially improve mental health issues, such as depression and anxiety (15), as well as improve metabolic health by influencing the composition of the gut microbiota (16). Recently, n-3 PUFA supplementation has been shown to slow down aging (17). In general, n-3 PUFAs are closely associated with health and longevity.

However, mammals, including humans, lack the ability to de novo synthesize n-3 PUFAs because they lack the genes for both the Δ12 desaturase enzyme, which converts oleic acid (C18:1n9) to linoleic acid (C18:2n6), and the Δ15 (n-3) desaturase enzyme, which converts n-6 PUFAs into n-3 PUFAs (18, 19). Therefore, humans need to obtain sufficient n-3 PUFAs from their diet or other nutritional supplements to meet health needs. In contrast, the model organism *Caenorhabditis elegans* possesses seven desaturases, FAT-1 to FAT-7, capable of de novo synthesis of PUFAs, including n-3 PUFAs (20, 21, 22, 23). Of these, FAT-1 is a unique desaturase converting n-6 PUFAs into n-3 PUFAs. The *C. elegans fat-1* gene has been successfully expressed in various organisms, including mice (24, 25), pigs (26), sheep (27), and fish, such as carp (28) and zebrafish (29), where it displayed the capability to increase the levels of n-3 PUFAs, including alpha-linolenic acid, EPA, and DHA. For example, *fat-1* transgenic mice showed improved metabolic health (24). In zebrafish, introduction of the transgene *Tg* (*CMV:fat1*) into the *elovl2* mutant background, or supplementing the diet with DHA, effectively alleviated the metabolic dysfunction-associated fatty liver disease and liver damage phenotypes of the *elovl2* mutants (29). Altogether, these works consistently demonstrated the potential applications of FAT-1 in improving metabolic health; however, the enzymatic activity and substrate specificity of FAT-1 remain unknown to date.

Integrating AlphaFold structure prediction, site-directed mutagenesis, and cross-species functional validation, we have identified the enzymatic and substrate-binding sites of *C. elegans* FAT-1. The amino acids G212, G216, and S217 were revealed to be required for FAT-1 activity. Moreover, FAT-1 displays a preference for converting n-6 PUFA substrate C20:4n6 (arachidonic acid) to C20:5n3 (EPA).

## Materials and methods

### Protein-small molecule docking experiment

A molecular docking study was undertaken to elucidate the binding affinity of the core compounds (ligands) to the proteins. The molecular structures (Mol2 structures) of the four n-6 fatty acids, including C18:2n6, C18:3n6, C20:3n6, and C20:4n6, were retrieved from PubChem (https://pubchem.ncbi.nlm.nih.gov/) (CID: 5280450, CID: 5280933, CID: 5280581, and CID: 444899). Chem 3D software was used to perform conformational optimization on each and was subsequently imported into AutoDockTools 1.5.7 for charge assignment (30, 31, 32, 33). Through hydrogenation and torsion angle detection, the structure was converted to pdbqt format. The predicted structure of FAT-1 (Q9NEQ0) was extracted from the protein structure database (https://www.uniprot.org). Protein FAT-1 was processed with hydrogenation and dehydration treatment, and its structure was converted to pdbqt format. The molecular docking of the protein and ligand was carried out in AutoDockTools 1.5.7, and visualizations were facilitated by the utilization of PyMOL software (Schrödinger, LLC).

### Mutagenesis design of FAT-1

In *C. elegans*, seven desaturases from FAT-1 to FAT-7 are involved in the biosynthesis of PUFAs (22, 23). Homology analysis on the protein sequences of these seven FAT desaturases showed that the homology percentages between FAT-1 and FAT-2, FAT-3, FAT-4, FAT-5, FAT-6, and FAT-7 were 52.83%, 18.10%, 17.21%, 14.45%, 17.18%, and 18.01%, respectively. Therefore, a specific amino acid (potential binding site to n-6 PUFAs) of FAT-1 was mutated to its corresponding amino acid in FAT-2 for function validation of FAT-1. In the case of when the amino acids at the corresponding sites of FAT-1 and FAT-2 were identical, the site was mutated to the corresponding amino acid in FAT-3 or FAT-4.

### *C. elegans* strains and maintenance

Worms were maintained on nematode growth medium plates seeded with *Escherichia coli* OP50, unless specifically indicated. WT N2 worms were purchased from the Caenorhabditis Genetic Center, and *fat-1(wa9)* worms were obtained from the Dr Jennifer L. Watts lab. All strains used in this study are listed in Supplemental Table S1.

Transgenic worms were created by microinjection following the methods previously described (34). In general, transgenic plasmids were constructed by homologous recombination. The *Pfat-1::fat-1::6× his::gfp* plasmid was generated by fusing *fat-1p::fat-1* (1.4 kb promoter and full-length *fat-1*), *6× his::gfp* (amplified from pPD95.77), and linearized pCFJ151 (amplified from pCFJ151). The *Pvha-6::fat-1::6× his::gfp* plasmid was generated by fusing *vha-6p* (1.3 kb promoter of *vha-6*), full-length *fat-1* without promoter, *6× his::gfp*, and linearized pCFJ151. The *Δ210-21*7, *Δ1-100*, *Δ103-351*, *G212M*, *G216L*, *S217R*, *Y90T*, *H168V-K171H*, *V176P-I178V*, *K190E*, *L210I*, *F211V*, *F213L*, *C214G*, *D215M*, *S230Q*, *W264Y*, and *L378M-D379N* plasmids were generated by fusing *vha-6p*, linearized FAT-1 with the indicated deletion/point mutations (amplified from *Pvha-6::fat-1::6× his::gfp*), and linearized pCFJ151. The primers used for the construction of all transgenes are listed in Supplemental Table S2.

Approximately 50 ng/μl transgenic plasmids, 50 ng/μl pJL43.1, and 2.5 ng/μl pCFJ90 were injected into the gonad of young adult *fat-1(wa9)* and EG4322 [*ttTi5605;unc-119(ed9)*] worms (35). Positive transgenic worms were selected based on fluorescence expression.

### Visualization of GFP

Late L4 worms expressing GFP were washed from nematode growth medium plates and settled in tubes. Then worms were mounted on an agarose pad (3%) and anesthetized with 100 nM levamisole. GFP fluorescence of entire worms was visualized under a Zeiss fluorescence microscope (Axio-Imager M2; Zeiss) equipped with an Axiocam 506 mono camera. At least 10 worms were observed for each condition in each biological repeat. Fluorescence images were captured using the same settings and exposure time for each nematode, unless specifically indicated. Images were processed and analyzed with ZEN 2 software (Zeiss).

### Cell culture

293T cells were cultured in DMEM (C11995500BT; Gibco) and supplemented with 10% fetal bovine serum (10099-141; Gibco) and 1% penicillin/streptomycin in 5% CO_2_ at 37°C. The culture medium was changed every 2 days unless otherwise indicated. The cell lines used in this study are listed in Supplemental Table S1.

### Construction of transgenic cells

FAT-1 was amplified from *C*. *elegans* complementary DNA. Full-length FAT-1 and specific site mutants (FAT-1(*G212M*), FAT-1(*G216L*), and FAT-1(*S217R*)) were subcloned into a *pCDH* lentiviral overexpression vector (with 3× FLAG). Lentiviral particles were produced by cotransfection of 293T cells with *pCDH*-FAT-1/FAT-1(*G212M*)/FAT-1(*G216L*)/FAT-1(*S217R*), psPAX2, and VSVG plasmids. After 48 h, the cell culture supernatant containing the packaged lentiviral particles was collected by centrifugation at 500 rpm for 5 min. The lentiviral particles were then infected into the target cell 293T with polybrene (107689; Sigma), ultimately yielding a stably transfected cell line. The stable cell lines were verified by DNA sequencing and Western blot analysis. The primers used for the construction of transgenes are listed in Supplemental Table S2.

### Fatty acid supplementation

n-6 fatty acids, including C18:2n6 (CDDE-U-59-A), C18:3n6 (CDAA-253189A), C20:3n6 (CDAA-253205M), and C20:4n6 (CDDE-U-71-A), were purchased from ANPEL. Fatty acids were dissolved in ethanol at a concentration of 10 mM and stored in a nitrogen environment until use (36). Cells were cultured with 10% FBS or DMEM for 72 h and cotreated with 40 μM of n-6 fatty acids or ethanol for the last 48 h. The final concentration of ethanol was 0.4%, which did not affect cell viability. Cells were collected for GC analysis to determine the catalytic efficiency of FAT-1 for different fatty acid substrates. The exogenous fatty acids used in this study are listed in Supplemental Table S3.

### GC detection

GC analysis was used to detect the contents of fatty acids in *C*. *elegans* and 293T cells. In brief, worms or cells were harvested on ice. For worms, 2,000 worms were washed with ddH_2_O and allowed to settle. For cells, 1 × 10^6^ cells were washed with cold PBS, scraped, and pelleted by centrifugation (1,000 *g*, 5 min). The supernatant was removed. The samples were then methylated using methanol containing 2.5% H_2_SO_4_ and heating for 2 h at 70°C. The methylated fatty acids were extracted by hexane and analyzed using an Agilent 8890A GC equipped with a 30 m × 0.25 mm × 0.25 μm DBWAX column, with nitrogen as the carrier gas at 1.4 ml/min, and a flame ionization detector (37). Three biological replicates were performed for each worm or cell strain. The 37-component fame mix (CDAA-M-252795-DZ; ANPEL) was used as the fatty acid standard to determine specific fatty acids. Percentages of each fatty acid were calculated as the ratio of the surface area of each considered peak to the total area of all peaks. If the percentage of fatty acid was lower than 0.3%, it was designated as undetectable in this study.

### Quantitative PCR analysis

Total RNA was isolated from worms following a previously described protocol (38). Complementary DNA was synthesized from 1 μg RNA using the PrimeScript RT reagent kit (RR047A; Takara Bio, Inc) following the manufacturer’s instructions. Quantitative PCR (qPCR) was performed using the SYBR Green Supermix (Bio-Rad) on a CFX96 Real-Time PCR System (Bio-Rad). Gene expression was quantified using the ΔΔCt method (39), and expression levels were normalized to tubulin as the housekeeping gene. Amplification conditions were as follows: an initial denaturation at 95°C for 3 min, followed by 40 cycles of denaturation at 95°C for 10 s, annealing at 56°C for 15 s, and extension at 72°C for 20 s. Melt curve analysis was performed to verify primer specificity. Data were analyzed using the Bio-Rad CFX Manager software. The qPCR primer sequences were as follows: Q*fat-1*F1: ATGGCAGAAAAGTCACAAGC; Q*fat-1*R1: TGGAACCCAATAGTACCAGA; Q*tbb-2*F1: TTTCACAAATGGGAGGAGGC; and Q*tbb-2*R1: GTCCACCCCAAAGCCAATC.

### Protein extraction and Western blot analysis

Western blot analysis was performed as described (34). Young adult worms were collected in H_2_O and washed three times on ice, then quickly frozen with liquid nitrogen, and stored at −80°C for later use. Worms were lysed with 100 μl RIPA supplemented with protease inhibitor cocktail (P8340; Sigma) and homogenized at 4°C. Cells were washed with cold PBS three times and lysed with RIPA buffer supplemented with a protease inhibitor cocktail. Protein concentrations were determined by BCA assay (23225; Thermo Fisher). Clarified lysates were denatured in 2× SDS buffer (100 mM Tris-HCl [pH 6.8], 20% glycerol, 4% SDS, 0.2% bromophenol blue, and 3% DTT) at 95°C for 5 min, equal amounts of protein samples were loaded and separated by 10% SDS-PAGE, and transferred to PVDF membranes at 0.4 A for 100 min (40). The membranes were blocked in 5% fat-free milk (Sangon Biotech; A600669) and then incubated with primary antibodies, including rabbit anti-GFP (1:1,000 dilution, HY-P80141; MCE), mouse anti-FLAG (1:1,000 dilution, F3165; Sigma-Aldrich), mouse anti-GAPDH (1:5,000 dilution, 60004-1-Ig; Proteintech), or mouse anti-β-actin (1:1,000 dilution, 66009-1-IG; Proteintech) at 4°C overnight. The membranes were washed and incubated with secondary antibodies, including goat anti-rabbit (1:5,000 dilution, 31460; Invitrogen) or goat anti-mouse (1:5,000 dilution, A24512; Thermo Fisher Scientific). Signal was detected using Image Quant LAS4000 (GE Healthcare) or SageCapture systems and analyzed with LANE 1D software (Sage Creation, Inc.). The antibodies used in this study are listed in Supplemental Table S3.

### Statistical analysis

All data are presented as SD ± mean. Statistical analysis of two groups of data was assessed by one-tailed Student's *t*-test, whereas data from more than two groups were analyzed by one-way ANOVA. *P* values less than 0.05 were considered statistically significant. Statistical analyses were performed using GraphPad Prism 8 (GraphPad Software, LLC) and Adobe Illustrator 2021 (Adobe, Inc.), unless otherwise stated.

## Results

### Prediction of potential substrate-binding amino acids of *C*. *elegans* FAT-1

**Fig. 1:**
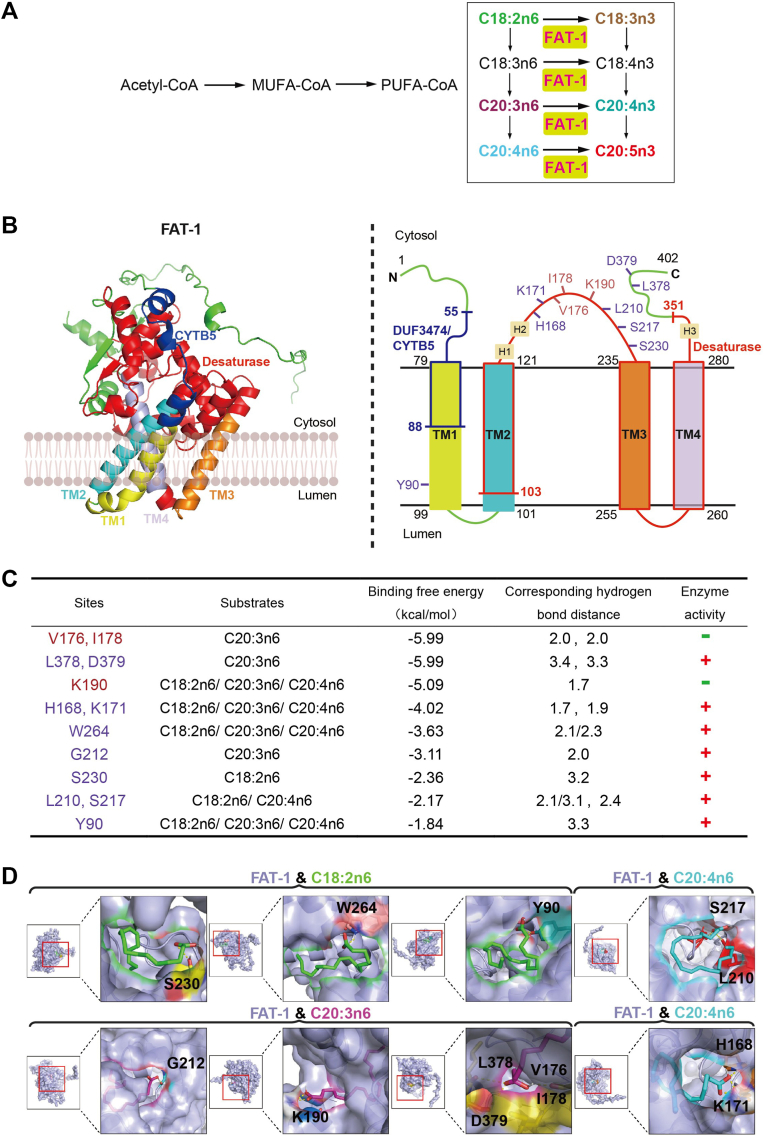
The function of FAT-1 in PUFA synthesis, its predicted structure, and its predicted binding sites with substrates. A: The biosynthetic pathway of PUFAs in *Caenorhabditis elegans*. B: The FAT-1 structure predicted by AlphaFold (left) and the corresponding topology model (right). Yellow, cyan, orange, and light purple helices (left) and boxes (right) represent the four TM region helices, respectively. The dark blue line and box indicate the DUF3474 domain (cytochrome b5-like domain). Red lines and boxes indicate the fatty acid desaturase region. Light yellow boxes H1, H2, and H3 represent conserved histidine residues. Different colors highlight the 11 potential binding sites of FAT-1 with three n-6 fatty acid substrates. C: Potential binding sites of FAT-1 to C18:2n6, C20:3n6, and C20:4n6 fatty acids, and the corresponding binding energies, hydrogen bonding distances, and results of whether mutations of this residue affect FAT-1 activity. D: Molecular docking results of FAT-1 with C18:2n6, C20:3n6, and C20:4n6 fatty acids. The figure shows the position of the pockets where the three substrates are predicted to interact with FAT-1 amino acid residues.

In *C. elegans*, the FAT-1 desaturase converts four n-6 PUFAs, including C18:2n6, C18:3n6, C20:3n6, and C20:4n6, into their corresponding n-3 PUFAs, C18:3n3, C18:4n3, C20:4n3, and C20:5n3, respectively (22, 23) (Fig. 1A). Because C18:4n3 is difficult to detect, as well as C18:3n3 and Δ19 fatty acid being indistinguishable by GC analysis, we chose to use the three n-6 PUFAs, C18:2n6, C20:3n6, and C20:4n6, to determine which amino acid(s) are important for the activity and substrate preference of the FAT-1 desaturase, as well as using AlphaFold for structure prediction and potential substrate molecular docking onto the FAT-1 protein.

The AlphaFold structure prediction showed that FAT-1 is predicted to be an integral membrane protein located in the endoplasmic reticulum (Fig. 1B), similar to the mouse or human stearoyl-CoA desaturase 1 (41, 42), with both the N terminus and C terminus of FAT-1 located on the cytoplasmic side of the membrane. FAT-1 has four transmembrane (TM) helices (TM1-4), each consisting of 21 amino acids, termed TM1 (AA79-99), TM2 (AA101-121), TM3 (AA235-255), and TM4 (AA260-280) (Fig. 1B). In addition, the FAT-1 desaturase domain contains 249 amino acids (103–351), including part of TM2 along with the full TM3 and TM4. Membrane-bound fatty acid desaturases from plants, animals, and other organisms contain three highly conserved histidine-rich sequences (His boxes) with the universal motifs HXXXH, HXXHH, and HXXHH. The three FAT-1 His boxes are termed H1 (AA123-127), H2 (AA159-163), and H3 (AA324-328) (Fig. 1B). These boxes are essential for enzyme activity (19). The DUF3474 domain, a predicated cytochrome b5 domain (43), spans from AA55 to AA88 (Fig. 1B). Molecular docking was able to identify a number of potential binding sites for at least one substrate. A number of the top-ranked binding sites were subsequently selected for functional validation based on the binding energies and corresponding hydrogen bond distances of these potential interacting amino acids (Fig. 1B–D).

### Overexpression of FAT-1 genetically complements an FAT-1 mutation in *C. elegans*

**Fig. 2:**
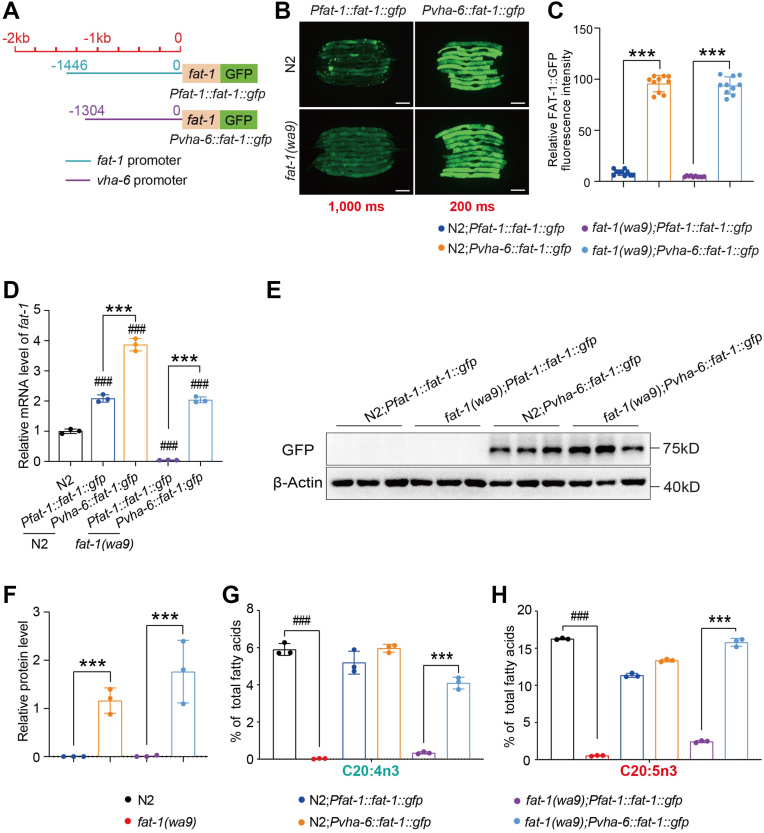
Overexpression of FAT-1 in *Caenorhabditis elegans* restores the function of the FAT-1 mutation. A: Schematic diagram of the transgenic *C. elegans* FAT-1::GFP sequence constructed using two promoters (the *fat-1* intrinsic promoter and the strong promoter *vha-6*). The blue solid line represents the *fat-1* intrinsic promoter sequence (1,446 bp upstream of coding sequence); the purple solid line represents the strong promoter *vha-6* sequence (1,304 bp upstream of coding sequence); the orange box denotes the coding region of FAT-1; the green box indicates the GFP label, and the GFP marker is located at the C terminus of FAT-1. B: Fluorescent intensity of FAT-1::GFP in worms driven by the *fat-1* intrinsic promoter and the strong promoter *vha-6*. The scale bar represents 100 μm. n = 10 for each worm strain. C: Quantification of fluorescence intensity in different worm strains in (B). D: The relative mRNA level of *fat-1* in transgenic worms. E: Protein level of FAT-1 in worms driven by the *fat-1* intrinsic promoter and strong promoter *vha-6*. F: Quantification of the protein levels of FAT-1 in worms driven by the *fat-1* intrinsic promoter and the strong promoter *vha-6*. G and H: Percentage of C20:4n3 (G) and C20:5n3 (H) of total fatty acids in worms driven by the *fat-1* intrinsic promoter and the strong promoter *vha-6*. All data are presented from three independent biological replicates. Significant difference between a specific mutant and WT N2, ^###^*P* < 0.001. Significant difference between the two indicated worm strains, ^∗∗∗^*P* < 0.001.

In *C. elegans*, the *fat-1(wa9)* mutation (Q156Stop) results in premature termination of the coding region, leading to a loss-of-function FAT-1 that is unable to synthesize n-3 PUFAs (22) (Supplemental Fig. S1). We therefore used the *fat-1(wa9)* mutant to test which amino acids are required for the substrate preference and activity of FAT-1. To do so, we needed to examine whether the WT *fat-1* gene could restore the missing n-3 PUFAs in the *fat-1(wa9)* mutant. First, we generated a *Pfat-1::fat-1::6× his::gfp* construct driven by the *fat-1* promoter (Fig. 2A), which was then introduced to both the WT (N2) worms and the *fat-1(wa9)* mutant worms by microinjection. However, the expression of *Pfat-1:*:FAT-1::6× HIS::GFP (simplified as *Pfat-1:*:FAT-1::GFP) was too weak to be detectable by either microscopic analysis of GFP fluorescence (Fig. 2B, C) or Western blot analysis with anti-GFP antibody (Fig. 2E, F). In addition, the level of *fat-1* mRNA was also confirmed by qPCR analysis (Fig. 2D). As well, GC analysis of the fatty acid profile showed that this construct had no effect on the levels of C20:4n3 and C20:5n3 in the *fat-1(wa9)* mutant (Fig. 2G, H and Supplemental Fig. S1).

We hypothesized that the failure of *Pfat-1:*:FAT-1::GFP to rescue the n-3 PUFAs in the *fat-1(wa9)* mutant worms might be due to weak expression when driven by its own promoter. We therefore used a stronger, somatic cell-specific promoter, *vha-6* (44). *Pvha-6::*FAT-1*::6× his::gfp* (simplified as *Pvha-6:*:FAT-1::GFP) showed much brighter GFP fluorescence, and the enhanced FAT-1 transcriptional and translational expression was confirmed by qPCR and Western blot in both the N2 and *fat-1(wa9)* mutant worms (Fig. 2B–F). More importantly, GC analysis revealed that the *Pvha-6:*:FAT-1::GFP expression could restore the n-3 PUFA C20:4n3 and C20:5n3 levels back to normal in the *fat-1(wa9)* mutant worms, while not increasing the n-3 PUFA levels in the WT worms (Fig. 2G, H and Supplemental Fig. S1). Taken together, these results demonstrate that sufficient overexpression of FAT-1 has the ability to restore the n-3 PUFA deficiency of the *fat-1(wa9)* mutant.

### Both the CYTB5/DUF3474 and desaturase domains are required for FAT-1 expression and activity

**Fig. 3:**
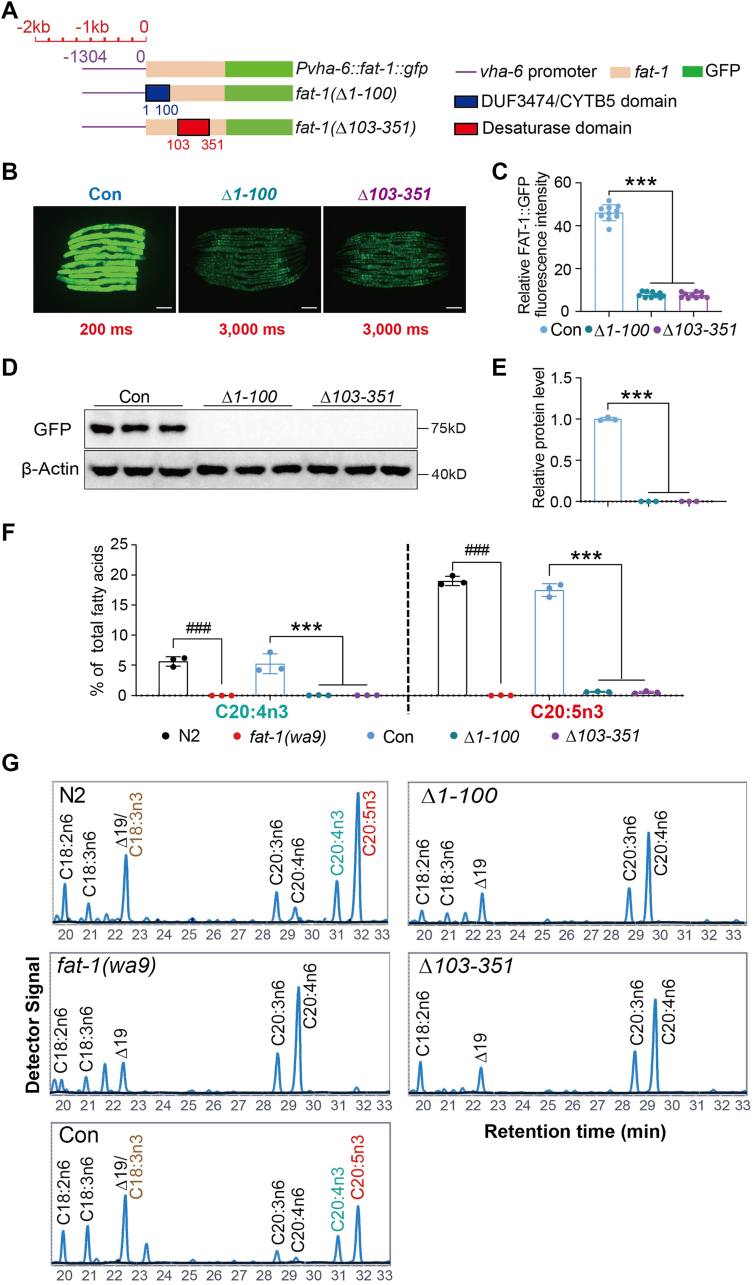
The DUF3474 domain and desaturase domain of FAT-1 affect FAT-1 expression and activity in *Caenorhabditis elegans*. A: Schematic diagram of FAT-1 truncation in *C. elegans*. Purple line: strong promoter *vha-6* sequence (1,304 bp upstream of coding sequence); orange box: coding sequence of FAT-1; green box: the GFP marker located at the C terminus of FAT-1; dark blue box: contains DUF3474/CYTB5 region truncation *Δ1-100*; red box: fatty acid desaturase region truncation *Δ103-351*. B: Fluorescent intensity of FAT-1::GFP in worms overexpressing WT, DUF3474 region deletion (*Δ1-100*), and fatty acid desaturase region (*Δ103-351*) deletion of FAT-1 protein. The scale bar represents 100 μm. C: Quantitation of the fluorescent intensity of worms in (B). n = 10 for each worm strain. D: Protein level of FAT-1 in worms overexpressing WT, DUF3474 region deletion (*Δ1-100*), and fatty acid desaturase region (*Δ103-351*) deletion of FAT-1 protein. E: Quantification of the protein levels of FAT-1 in (D). F: Percentage of C20:4n3 and C20:5n3 in total fatty acids for worms overexpressing WT, DUF3474 region deletion (*Δ1-100*), and fatty acid desaturase region (*Δ103-351*) deletion of FAT-1 protein. G: GC profile of PUFAs in different worm strains. All data are presented from three independent biological replicates. Significant difference between a specific mutant and WT N2, ^###^*P* < 0.001. Significant difference between the two indicated worm strains, ^∗∗∗^*P* < 0.001.

To activate desaturases, including FAT-1 to FAT-4, electrons are transferred from the cytochrome b5 reductase HPO-19/T05H4.4, thereby facilitating the synthesis of PUFAs (43). We hypothesized that the DUF3474 domain (AA55-88) in the N terminus of FAT-1 may function as a cytochrome b5 domain to receive electrons from the cytochrome b5 reductase (HPO-19/T05H4.4) and then pass them to its desaturase domain (AA103-351) in the C terminus for enzyme activation (Fig. 1B). However, it is unknown whether both domains are required for FAT-1 activity. We therefore constructed two truncation mutations, Δ1-100 and Δ103-351, producing deletion mutations without the DUF3474 domain and the desaturase domain, respectively. We then introduced these mutations into the *fat-1(wa9)* mutant worms (Fig. 3A). However, compared with the control (CON), the protein expression of both truncation mutants was too weak to be detected either by microscopic analysis of GFP fluorescence (Fig. 3B, C) or by Western blot analysis using anti-GFP antibody (Fig. 3D, E). Furthermore, GC analysis showed that, unlike the full-length *Pvha-6::fat-1::gfp* construct, neither truncation mutation could restore the n-3 PUFA levels in *fat-1(wa9)* mutant worms (Fig. 3F, G), indicating that both domains are essential for FAT-1 expression and activity.

### Several amino acids are critical for the activity and protein level of FAT-1

**Fig. 4:**
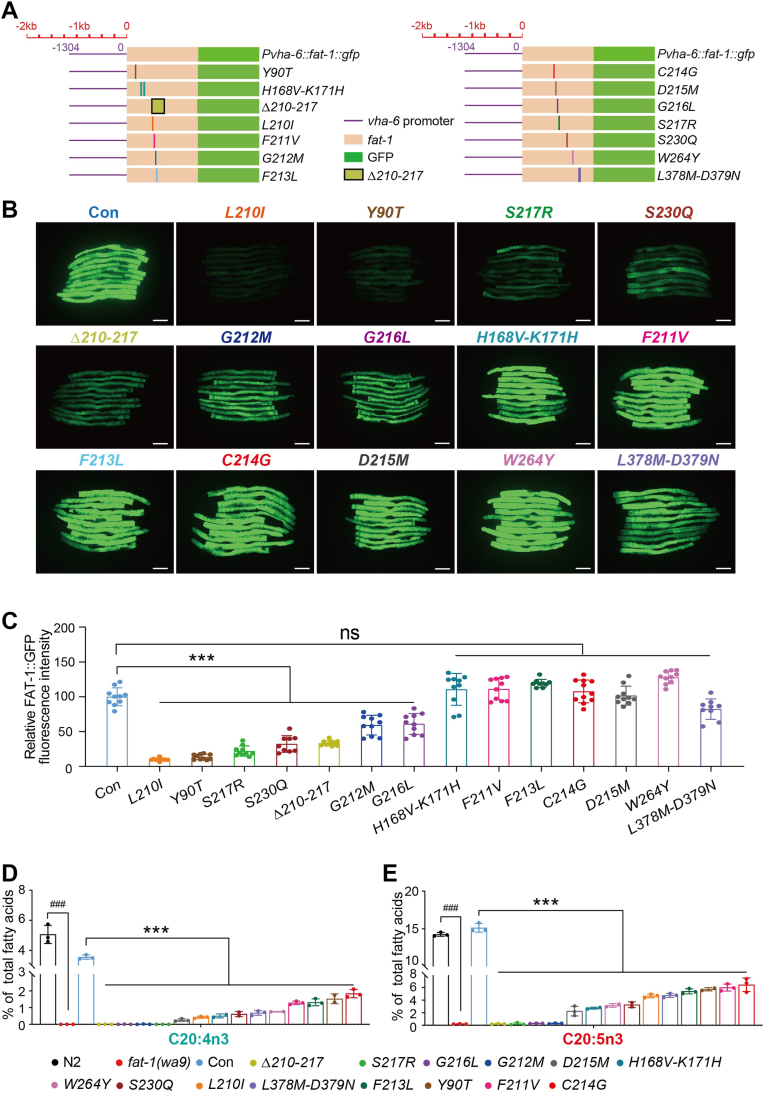
Point mutations of FAT-1 significantly reduce its activity in *Caenorhabditis elegans*. A: The schematic diagrams of FAT-1 point mutation/deletion in *C. elegans*. Purple line: strong promoter *vha-6* sequence (1,304 bp upstream of coding sequence); orange box: coding sequence of FAT-1; green box: the GFP marker located at the C terminus of FAT-1; and yellow boxes: *Δ210-217* truncation. The solid line in each diagram indicates a specific site mutation, and the colors in the diagrams correspond to the colors for worm strains in (B). B: Fluorescent intensity of FAT-1::GFP in different FAT-1 point mutation or deletion worms. The scale bar represents 100 μm. C: Quantitation of the fluorescent intensity of worms in (B). n = 10 for each worm strain. D and E: Percentage of C20:4n3 (D) and C20:5n3 (E) in total fatty acids for different worm strains. All data are presented from three independent biological replicates. Significant difference between a specific mutant and WT N2, ^###^*P* < 0.001. Significant difference between the two indicated worm strains, ^∗∗∗^*P* < 0.001, ns, no significance.

We next used the *fat-1(wa9);Pvha-6::fat-1::gfp* system to examine which amino acids in the predicted substrate-binding domains determine the activity of FAT-1. We generated worms containing mutations in the top-ranked potential binding sites using the MosSCI method (35) (Figs. 1B, C and 4A) and then detected protein expression by GFP fluorescence and Western blot analysis as well as fatty acid profiles by GC analysis. For convenience, the *fat-1(wa9);Pvha-6::fat-1::gfp* line was simplified as CON, and each of the mutations was named as the mutated amino acid in FAT-1 (Fig. 4A).

Amino acids 210–217 contain several potential binding sites, such as G212 binding to C20:3n6 and L210 and S217 binding to C18:2n6 and C20:4n6 (Fig. 1C, D). Therefore, we first generated a deletion mutation line, *fat-1(wa9);Pvha-6::fat-1(Δ210-217)::gfp* (Fig. 4A). Deletion of amino acids 210–217 led to significantly reduced expression of FAT-1::GFP (Fig. 4B, C) and also failed to restore the levels of n-3 PUFAs C20:4n3 and C20:5n3 in the *fat-1(wa9)* mutant worms (Fig. 4D, E and Supplemental Fig. S2), suggesting that this region is necessary for the activity of FAT-1.

To determine which specific amino acids in this region are required for the activity of FAT-1, we used the same strategy and technology to generate *L210I*, *F211V*, *G212M*, *F213L*, *C214G*, *D215M*, *G216L*, and *S217R* individual point mutation worms (Fig. 4A). Similar to the Δ210-217 deletion worms, mutation of *G212M*, *G216L*, or *S217R* led to complete loss of *Pvha-6*::FAT-1::GFP function in the *fat-1(wa9)* mutant worms (Fig. 4D, E and Supplemental Fig. S2) and also reduced the FAT-1::GFP expression to different extents (Fig. 4B, C). In addition, although the mutation of *F211V*, *F213L*, *C214G*, or *D215M* did not affect the FAT-1::GFP protein levels, they did display reduced levels of C20:4n3 and C20:5n3 to different extents (Fig. 4B–E and Supplemental Fig. S3).

Based on the results of molecular docking analysis, several amino acids, such as Y90, H168-K171, V176-I178, K190, S230, W264, and L378-D379, were also selected for functional validation (Figs. 1B, C, 4A and Supplemental Fig. S4). The same strategy as mentioned above was used to generate their corresponding mutations (Fig. 4A and Supplemental Fig. S4A). Similar to *L210I*, mutation of *Y90T* reduced the expression of FAT-1::GFP the most (Fig. 4B, C), and both mutations also affected the FAT-1 conversion of C20:4n3 and C20:5n3 (Fig. 4D, E and Supplemental Fig. S3). Mutation of *H168V-K171H*, *W264Y*, or *L378M-D379N* significantly reduced the levels of C20:4n3 and C20:5n3, while not affecting the protein levels of FAT-1::GFP (Fig. 4B–E and Supplemental Fig. S3). In contrast, mutation of either *V176P-I178V* or *K190E* had no effects on both the protein level and activity of FAT-1 (Supplemental Figs. S4A–E and S3). Collectively, six point mutations: *Y90T*, *L210I*, *G212M*, *G216L*, and *S217R* plus the Δ210-217 deletion affect both FAT-1 protein level and activity; while mutations of *F211V*, *F213L*, *C214G*, *D215M*, *H168V-K171H*, *W264Y*, or *L378M-D379N* affect only FAT-1 activity but not the protein level.

### Substrate preference of FAT-1 in mammalian cells

**Fig. 5:**
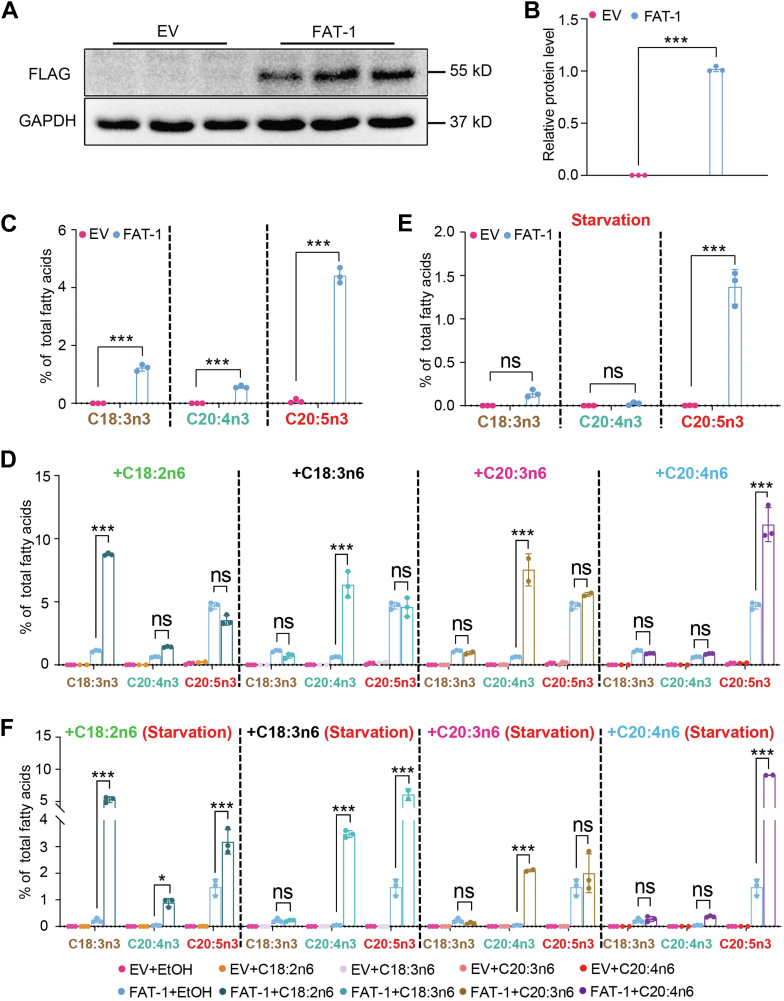
FAT-1 converts n-6 PUFAs to n-3 PUFAs in a mammalian cell system. A: The protein levels of FAT-1 in CON cells (*pCDH*, empty vector, simplified as EV) and transgenic FAT-1 cells (*pCDH-*FAT-1*::FLAG,* simplified as FAT-1). B: Quantification of the protein levels of FAT-1 in (A). C: Percentage of C18:3n3, C20:4n3, and C20:5n3 in total fatty acids for different cells under normal dietary conditions. D: Percentage of C18:3n3, C20:4n3, and C20:5n3 in total fatty acids for different cells supplemented with C18:2n6, C18:3n6, C20:3n6, and C20:4n6 substrates, respectively, under normal dietary conditions. E: Percentage of C18:3n3, C20:4n3, and C20:5n3 of total fatty acids for different cells under starvation conditions. F: Percentage of C18:3n3, C20:4n3, and C20:5n3 of total fatty acids for different cells supplemented with C18:2n6, C18:3n6, C20:3n6, and C20:4n6 substrates, respectively, under starvation conditions. All data are presented from three independent biological replicates. Significant difference between the two indicated cell lines, ^∗^*P* < 0.05, ^∗∗∗^*P* < 0.001, ns, no significance.

In *C*. *elegans*, FAT-1 converts four n-6 PUFAs, C18:2n6, C18:3n6, C20:3n6, and C20:4n6, into their corresponding n-3 PUFAs (Fig. 1A), raising the question as to which specific n-6 PUFA is the most preferred substrate for FAT-1. Since *C*. *elegans* can de novo synthesize all n-6 PUFAs and n-3 PUFAs via the FAT-1 to FAT-4 desaturases (22, 23), it is difficult to examine the substrate preference of FAT-1 by diet supplementation of a specific n-6 PUFA. Therefore, we opted to use mammalian 293T cells. We first generated a cell line stably expressing FAT-1 (*pCDH-*FAT-1*::3× FLAG,* simplified as FAT-1). Western blot analysis using anti-FLAG antibody showed that FAT-1 was successfully expressed in 293T cells (Fig. 5A, B). Next, these transgenic cells, along with the CON cells (*pCDH*, empty vector), were harvested to analyze the fatty acid profile by GC analysis. The n-3 PUFAs, C18:3n3, C20:4n3, and C20:5n3, were undetectable in 293T cells, whereas overexpression of FAT-1 showed their presence (Fig. 5C and Supplemental Fig. S5A). Compared with the levels of C18:3n3 and C20:4n3, the level of C20:5n3 was the highest (Fig. 5C and Supplemental Fig. S5A), similar to what was observed in *C. elegans* (Supplemental Fig. S1).

Next, to test the ability of FAT-1 to convert a specific n-6 PUFA to its corresponding n-3 PUFA, we individually added exogenous C18:2n6, C18:3n6, C20:3n6, or C20:4n6 to the *pCDH-*FAT-1 cells. After 48 h of treatment, cell samples were collected and analyzed by GC to determine the levels of n-3 PUFAs. GC analysis revealed that exogenous addition of C18:2n6, C20:3n6, or C20:4n6 significantly increased the corresponding production of C18:3n3, C20:4n3 or C20:5n3, respectively, but did not affect other n-3 PUFAs (Fig. 5D and Supplemental Fig. S5B). Interestingly, exogenous addition of C18:3n6 increased the level of downstream n-3 PUFA C20:4n3 (Fig. 5D and Supplemental Fig. S5B).

We also questioned if n-6 PUFAs present in the cell culture medium could consequently affect the substrate preference of FAT-1 and also its conversion capacity on different substrates. To test this hypothesis, the cells were cultured in DMEM without FBS (starvation condition) for 24 h, exogenous n-6 PUFAs were added to the DMEM for another 48 h, and finally, the cells were harvested for the fatty acid profile GC analysis. Under the starvation condition, only C20:5n3 was detectable from cells overexpressing FAT-1 as compared with the CON (empty vector) cells (Fig. 5E and Supplemental Fig. S6A), implying that FAT-1 may prefer to produce C20:5n3 first. However, consistently, under starvation, FAT-1 could still convert exogenous C18:2n6, C18:3n6, C20:3n6, and C20:4n6 into their corresponding and downstream n-3 PUFAs (Fig. 5F and Supplemental Fig. S6B). Taken together, these results confirm that the FAT-1 has the capacity to convert all four n-6 PUFAs to n-3 PUFAs in both *C. elegans* and mammalian cells.

### Amino acids G212, G216, and S217 are also essential for FAT-1 activity in mammalian cells

**Fig. 6:**
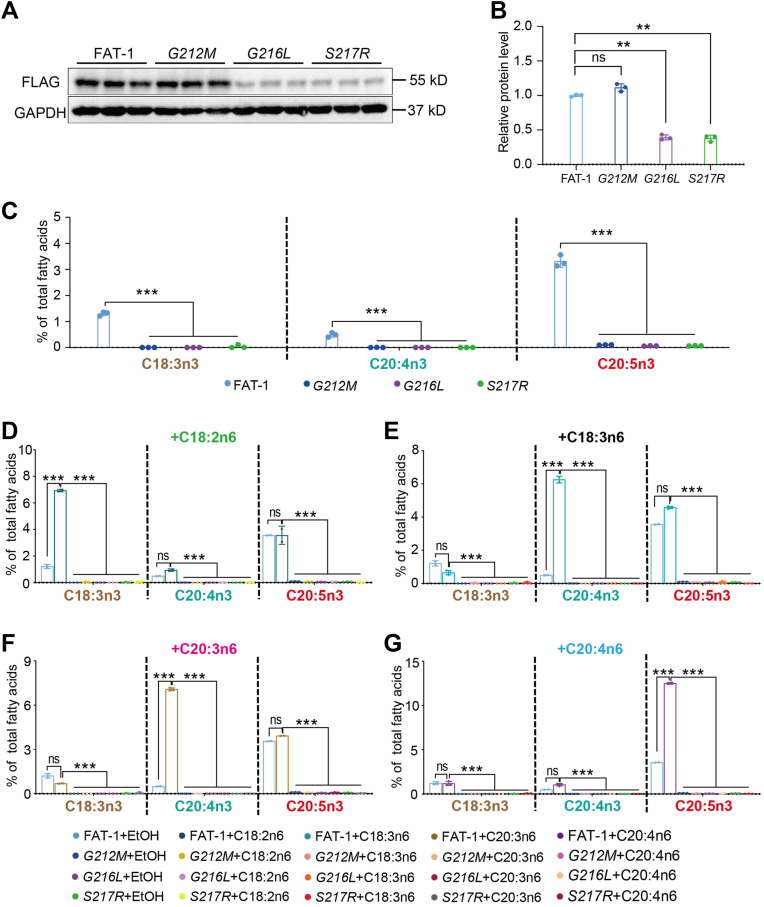
FAT-1 point mutations display reduced activity in mammalian cells. A: The protein levels of FAT-1 in its CON (FAT-1) and transgenic overexpression cells containing point mutations. B: Quantification of the protein levels of FAT-1 proteins in (A). C: Percentage of C18:3n3, C20:4n3, and C20:5n3 in total fatty acids for CON and FAT-1 point mutation transgenic cells under normal dietary conditions. D-G: Percentage of C18:3n3, C20:4n3, and C20:5n3 in total fatty acids for different transgenic cells supplemented with C18:2n6 (D), C18:3n6 (E), C20:3n6 (F), and C20:4n6 (G) substrates under normal dietary conditions. All data are presented from three independent biological replicates. Significant difference between the two indicated cell lines, ^∗∗^*P* < 0.01, ^∗∗∗^*P* < 0.001, ns, no significance.

As mentioned above, amino acids G212, G216, and S217 are required for the activity of FAT-1 in *C. elegans*. We tested their function in 293T cells by generating stably expressing cell lines based on *pCDH-*FAT-1*::FLAG* with the individual point mutations. Western blots showed that both the *G216L* and *S217R* point mutations reduced the protein levels of FAT-1, but in 293 cells, the *G212M* mutation did not (Fig. 6A, B). GC analysis revealed that all three mutations in FAT-1 did not produce the n-3 PUFAs, C18:3n3, C20:4n3, and C20:5n3 (Fig. 6C and Supplemental Fig. S7). In addition, the mutants could not convert exogenous C18:2n6, C18:3n6, C20:3n6, or C20:4n6 into their corresponding n-3 PUFAs under normal (Fig. 6D–G) and starvation conditions (Supplemental Fig. S8A–E). Thus, these observations were consistent with the results in *C. elegans*, further confirming the essential role of these amino acids for FAT-1 activity.

## Discussion

FAT-1 is the desaturase that specifically converts n-6 PUFAs into n-3 PUFAs (22, 23). It has been successfully expressed in various animals to increase n-3 PUFA production (24, 26, 27, 28, 29). In this study, several amino acids have been found to be required for the activity of FAT-1 in both *C. elegans* and 293T mammalian cells. In particular, deletion of L210-S217 or mutating G212, G216, and S217 in FAT-1 led to complete loss of the conversion of n-6 PUFAs to n-3 PUFAs, suggesting that this region is critical for its activity.

Of all the n-3 PUFAs in *C. elegans*, C20:5n3 constitutes the largest pool. In mammalian cells, overexpression of FAT-1 also resulted in a higher level of C20:5n3 than C18:3n3 and C20:4n3, especially under a starvation condition, where C20:5n3 was the only detectable n-3 PUFA. In addition, transgenic expression of FAT-1 in mice, pigs, sheep, and fish also showed a consistent increase of C20:5n3 in all these species (24, 26, 27, 28). Thus, FAT-1 may have a preference to produce C20:5n3 via the conversion from C20:4n6 relative to other n-6 PUFAs.

Supplementing specific n-6 PUFAs in *C. elegans* to validate the conversion capacity of FAT-1 was challenging. Therefore, an FAT-1 stable cell line was constructed, and FAT-1 was found to be capable of converting n-6 PUFAs into n-3 PUFAs. In addition, to test the conversion capacity of FAT-1, we added exogenous C18:2n6, C18:3n6, C20:3n6, or C20:4n6 under normal and starved conditions and found that all four n-6 PUFAs could be converted into their corresponding n-3 PUFAs or downstream n-3 PUFAs without affecting the generation of upstream n-3 PUFAs, and this ability was also effective in *C. elegans*. Although the GC technology cannot detect C18:4n3 in our study, dietary supplementation of C18:3n6 led to increased levels of downstream fatty acids C20:4n3 and C20:5n3 in mammalian cells, supporting that the FAT-1 has the ability to convert C18:3n6 to C18:4n3, which is further used to produce C20:4n3 and C20:5n3. Thus, the FAT-1 desaturase indeed has a function for the conversion of all four n-6 PUFAs.

Sequence analysis revealed that FAT-1 possesses a DUF3474 domain in the N terminus, which may be a cytochrome b5-like domain, and a desaturase domain in the C terminus (43). However, the function of these predicted domains was previously unknown. In this study, deletion of either domain (Δ1-100 and Δ103-351) resulted in a significantly reduced FAT-1 protein expression and a complete loss of n-3 PUFAs, confirming their indispensable role for the biosynthesis of PUFAs. Whether the loss of activity of both truncated FAT-1 was due to their low protein expression needs future investigation. Moreover, the majority of amino acids that affect FAT-1 activity, such as S217, G212, and G216, are located on the cytoplasmic side of the desaturase domain. As well, the *G216L* and *S217R* point mutations exhibited consistently reduced protein levels in both *C. elegans* and mammalian cells to different extents. However, the *G212M* point mutation significantly decreased the protein level of FAT-1 in *C. elegans* but not in mammalian cells. This discrepancy may result from species differences or other unknown factors affecting the protein stability, which needs future investigation.

**Fig. 7:**
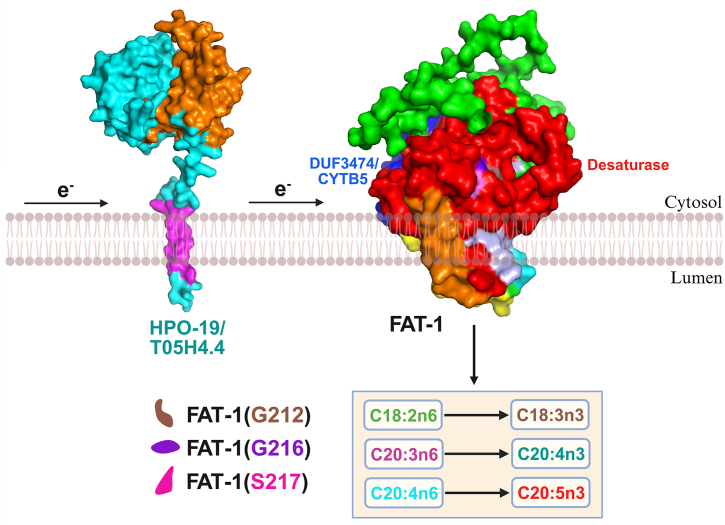
Proposed molecular mechanism of FAT-1 in n-3 PUFAs biosynthesis. Cytochrome b5 reductase HPO-19 first transfers electrons to the DUF3474 domain of fatty acid desaturase FAT-1. These electrons then move to the active site of the desaturase, thereby activating it for n-6/n-3 fatty acid conversion. However, when FAT-1 is functionally defective (*G212M/G216L/S217R* point mutations), n-3 PUFA synthesis is impaired, leading to an n-6/n-3 metabolic imbalance. Brown shape: location of point mutation G212; purple shape: location of point mutation G216; and pink shape: location of point mutation S217. Image was created with BioRender.com.

We previously reported that cytochrome b5 reductase HPO-19/T05H4.4 is required for the desaturation reaction for PUFA biosynthesis in *C. elegans* (43). Based on this previous study, and our presented data, we propose that electrons pass from cytochrome b5 reductase HPO-19/T05H4.4 to the DUF3474/CYTB5 domain of FAT-1 and then to its desaturase domain to activate n-3 PUFA biosynthesis (Fig. 7). Unraveling this mechanism is an area for further research.

## Data Availability

The data that support the findings of this study are available from the corresponding authors upon reasonable request.

## Supplemental data

This article contains supplemental data.

## Conflict of interest

The authors declare that they have no conflicts of interest with the contents of this article.

## Figures and Tables

**Revised Figure S1:**
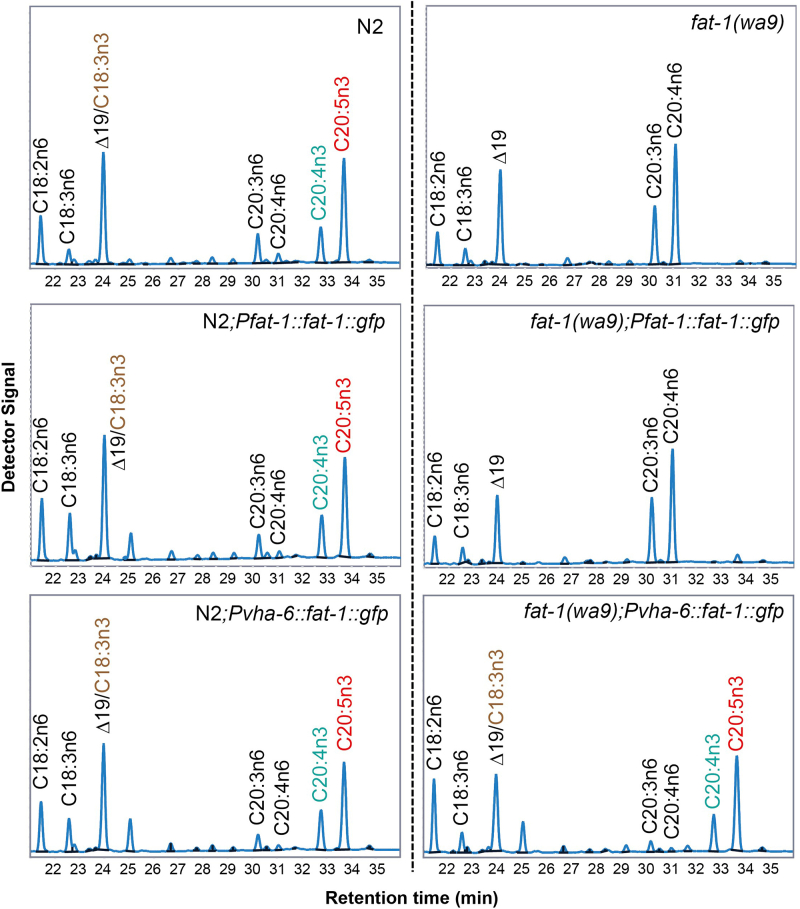
Gas chromatography (GC) profiles of PUFAs in N2 and *fat-1(wa9)* worms.

**Revised Figure S2:**
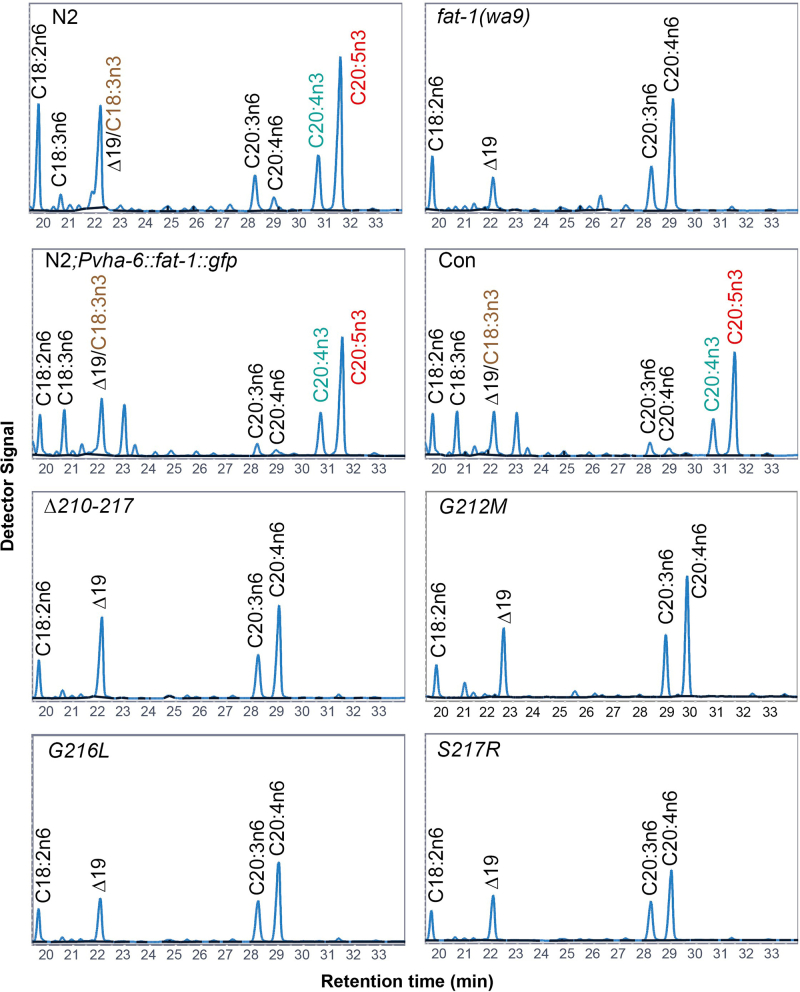
The AAs 210-217, G212, G216, and S217 in FAT-1 are required for FAT-1 activity analyzed by GC profile. The *fat-1(wa9);Pvha-6::fat-1::gfp* was simplified as Con.

**Revised Figure S3:**
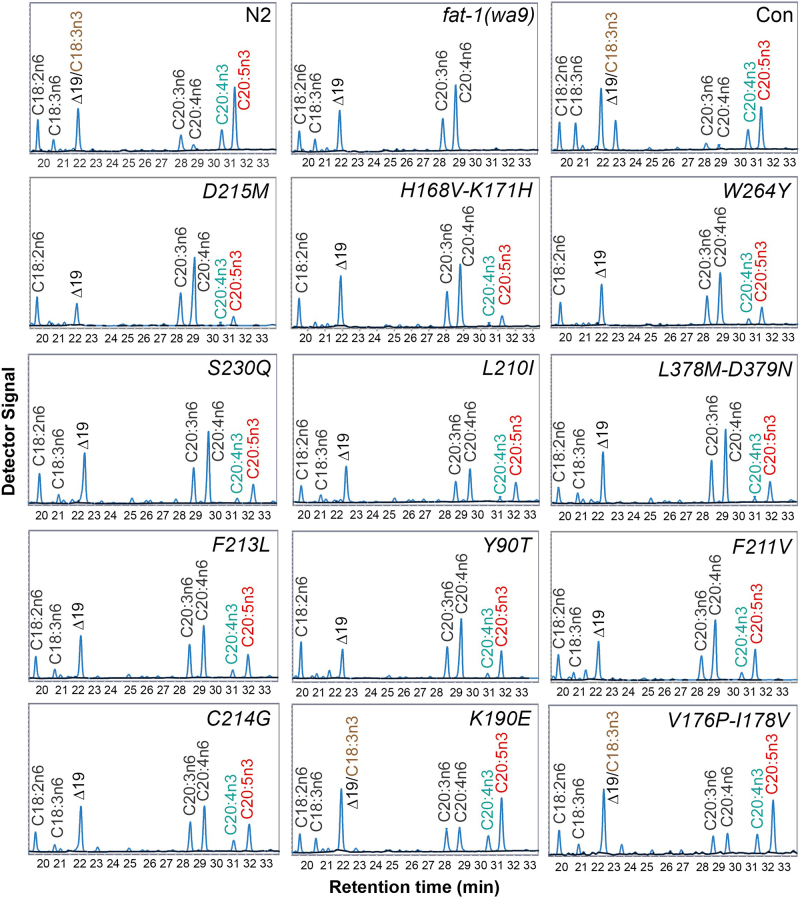
GC profiles for various mutants. The *fat-1(wa9);Pvha-6::fat-1::gfp* was simplified as Con.

**Revised Figure S4:**
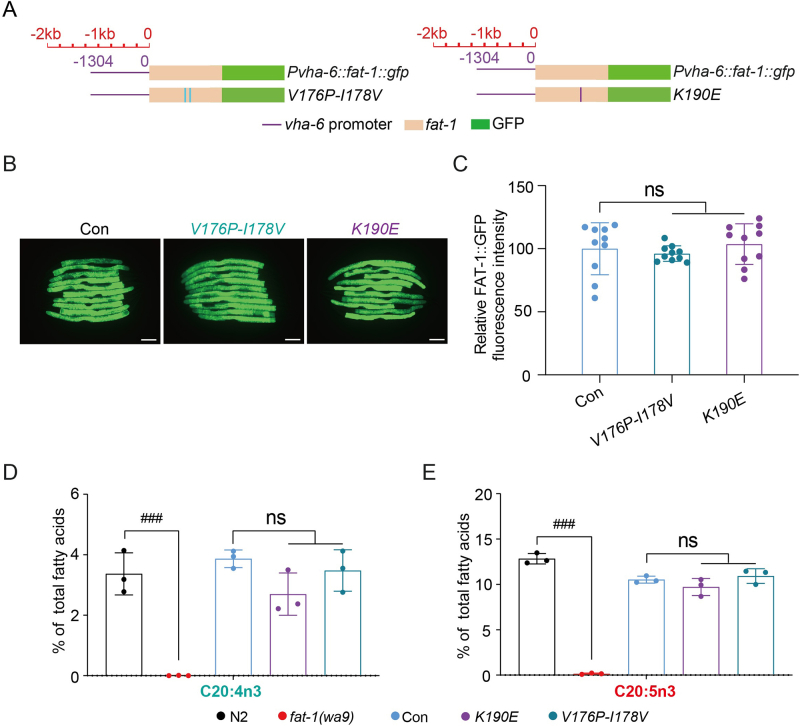
The AAs V176-I178 and K190 point mutations do not affect the activity of FAT-1. A: Schematic diagrams of these FAT-1 point mutations in *C. elegans*. Purple line: strong promoter *vha-6* sequence (1,304 bp upstream of coding sequence); Orange box: coding sequence of FAT-1; Green box: the GFP marker located at the C-terminus of FAT-1; The solid line in each diagram indicates a specific site mutation, and the colors in the diagrams correspond to the colors for the worm strains in (B). B: Fluorescent intensity of FAT-1::GFP in different FAT-1 point mutation worms. Scale bar represents 100 μm. C: Quantitation of the fluorescent intensity of worms in (B). n = 10 for each worm strain. D and E: Percentage of C20:4n3 (D) and C20:5n3 (E) of total fatty acids for different worm strains. All data are presented from three independent biological replicates. Significant difference between a specific mutant and wild type N2, ^^###^^*P* < 0.001. Significant difference between two indicated worm strains, ns, no significance.

**Revised Figure S5:**
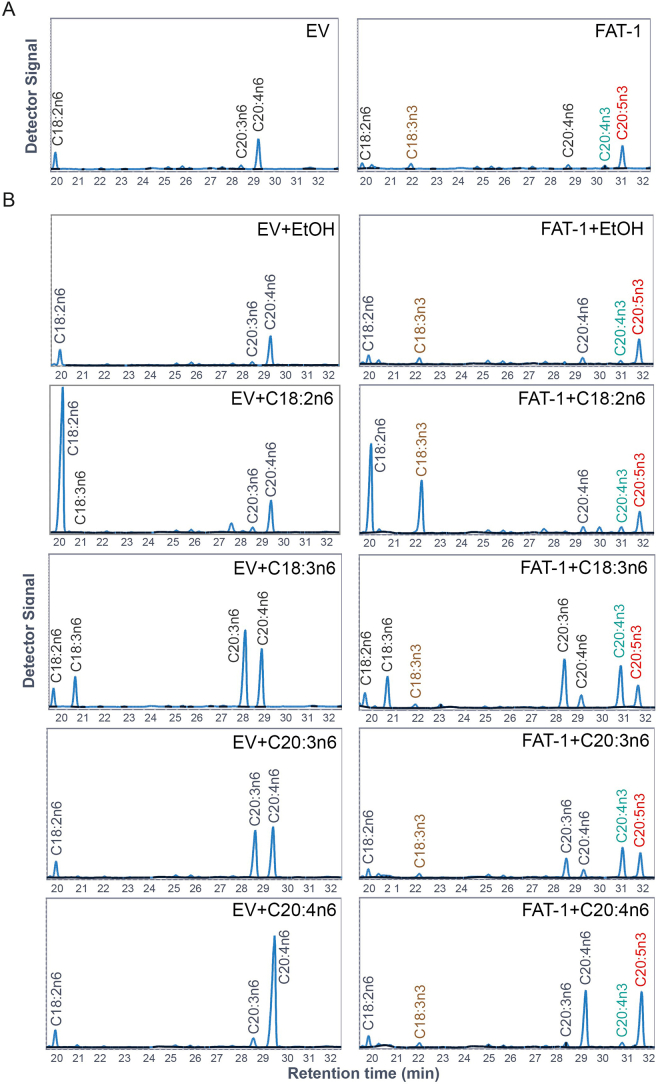
Supplementation of n-6 PUFAs significantly increased their corresponding or downstream products as analyzed by GC under normal dietary conditions. A: GC profile of PUFAs in EV and transgenic wild type FAT-1 (simplified as FAT-1) cells under normal dietary conditions. B: GC profile of PUFAs in EV and transgenic wild type FAT-1 (simplified as FAT-1) cells treated with four n-6 fatty acids, respectively, under normal dietary conditions.

**Revised Figure S6:**
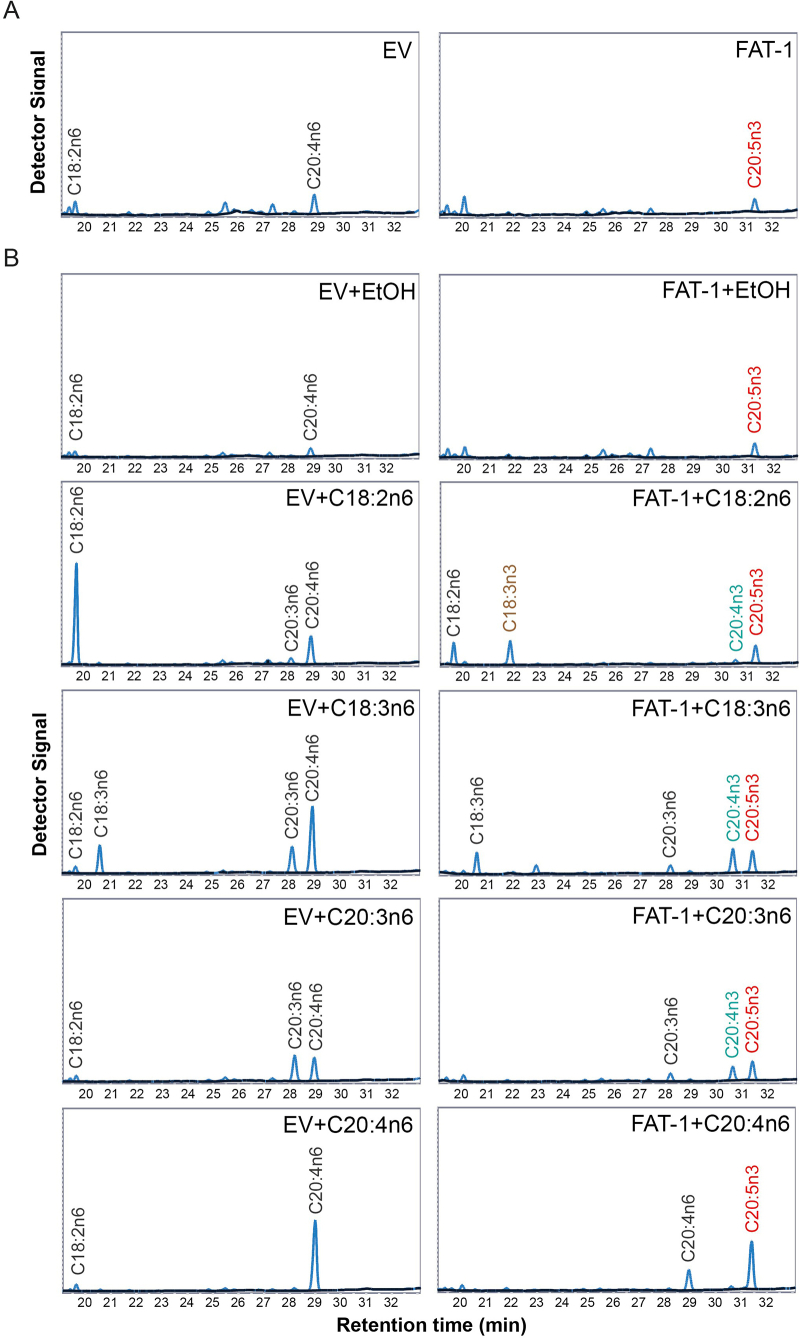
Exogenous supplementation of n-6 PUFAs could induce the production of n-3 PUFAs under starvation conditions in mammalian cells analyzed by GC. A: GC profile of PUFA in EV and transgenic wild type FAT-1 (simplified as FAT-1) cells under starvation conditions. B: GC profile of PUFA in EV and transgenic wild type FAT-1 (simplified as FAT-1) cells treated with four n-6 fatty acids, respectively, under starvation conditions.

**Revised Figure S7:**
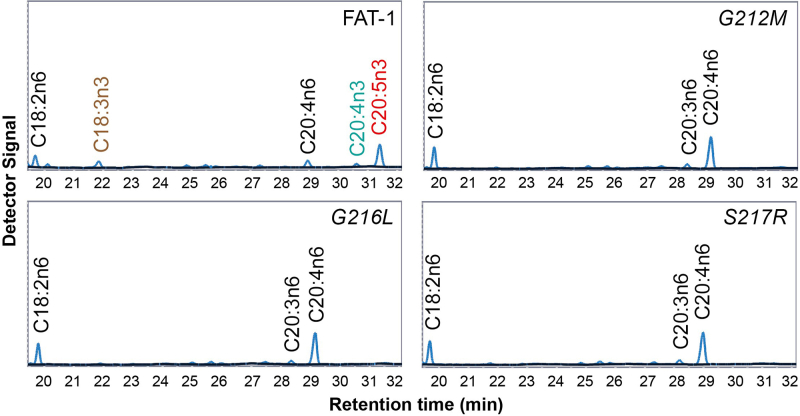
The AAs G212, G216 and S217 are required for FAT-1 activity analyzed by GC profile in cells with transgenic wild type FAT-1 (simplified as FAT-1) or transgenic point mutation FAT-1.

**Revised Figure S8:**
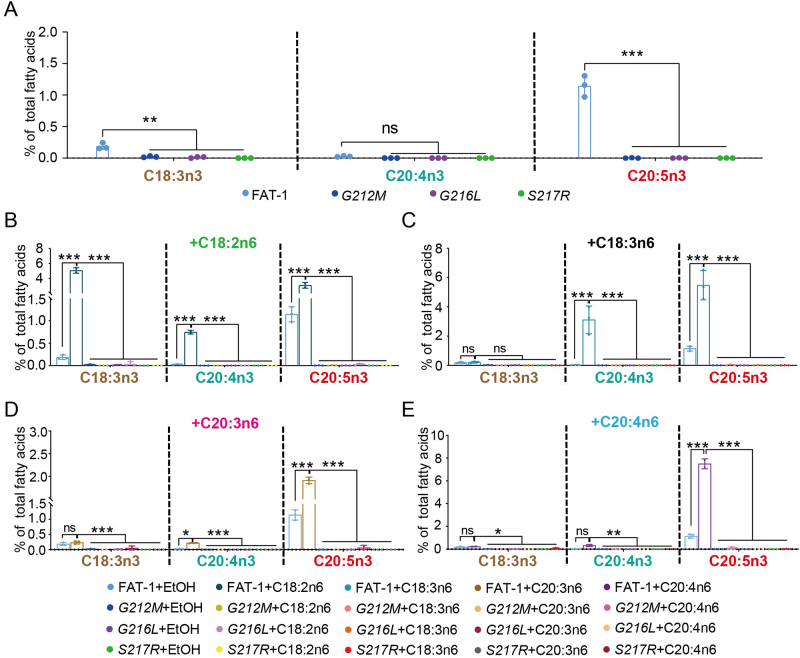
The AAs 210-217 in FAT-1 are required for FAT-1 activity analyzed by GC profile in cells with transgenic wild type or point mutation transgenic FAT-1 under starvation conditions. A: Percentage of C18:3n3, C20:4n3, and C20:5n3 of total fatty acids for transgenic wild type FAT-1 (simplified as FAT-1) and point mutation transgenic cells under starvation conditions. B–E: Percentage of C18:3n3, C20:4n3, and C20:5n3 of total fatty acids for FAT-1 wild type and point mutation transgenic cells treated with C18:2n6 (B), C18:3n6 (C), C20:3n6 (D), C20:4n6 (E), respectively, under starvation conditions. All data are presented from three independent biological replicates. Significant difference between two indicated worm strains, ^∗^*P* < 0.05, ^∗∗^*P* < 0.01, ^∗∗∗^*P* < 0.001, ns, no significance.

## Supplementary Data

Revised Supplementary tables
