# Supplementary material for: Determination of FAT-1 desaturase activity and substrate preference
Source: J Lipid Res. 2025 Nov 14;66(12):100945. doi: 10.1016/j.jlr.2025.100945 (PMC12743521; doi:10.1016/j.jlr.2025.100945)
Supplement: Revised Supplementary tables [file mmc1.docx]

**Supplemental Table S1 Biological materials used in this study.**

| ***C. elegans* strains** | | | | | | |
| --- | --- | --- | --- | --- | --- | --- |
| **Strain name** | **Short name in this study** | **Genotype** | | | **Source** | |
| N2 |  | N2 (Wild Type) | | | CGC | |
| BX24 |  | *fat-1(w*a*9) IV* | | | Jennifer L. Watts lab | |
| EG4322 |  | *ttTi5605 II; unc-119(ed9) III.* | | | Shouhong Guang lab | |
| Lib2222 | N2;Pfat-1::fat-1::gfp | *unc-119(ed9);k*unEx321[Pfat-1::fat-1::6*×* His-tag::gfp *+ unc-119(+)*] | | | This study | |
| Lib2233 | *fat-1(w*a*9)*;Pfat-1::fat-1::gfp | *fat-1(w*a*9);k*unEx321[Pfat-1::fat-1::6*×* His-tag::gfp *+ unc-119(+)*] | | | This study | |
| Lib2220 | N2;Pvha-6::fat-1::gfp | *unc-119(ed9);kunEx317[Pvha-6::fat-1::6× His-tag::gfp + unc-119(+)]* | | | This study | |
| Lib2242 | Con | *fat-1(w*a*9);kunEx317[Pvha-6::fat-1::6× His-tag::gfp + unc-119(+)]* | | | This study | |
| Lib2683 | *∆1-100* | *fat-1(wa9); kunEx322[Pvha-6::fat-1(∆1-100 aa)::6× His-tag::gfp + unc-119(+)]* | | | This study | |
| Lib2995 | *∆103-351* | *fat-1(wa9); kunEx323[Pvha-6::fat-1(∆103-351 aa)::6× His-tag::gfp + unc-119(+)]* | | | This study | |
| Lib2414 | *Y90T* | *fat-1(wa9); kunEx324[Pvha-6::fat-1(Y90T)::6× His-tag::gfp + unc-119(+)]* | | | This study | |
| Lib2436 | *V176P-I178V* | *fat-1(wa9); kunEx325[Pvha-6::fat-1(V176P-I178V)::6× His-tag::gfp + unc-119(+)]* | | | This study | |
| Lib2476 | *∆210-217* | *fat-1(wa9); kunEx326[Pvha-6::fat-1(∆210-217 aa)::6× His-tag::gfp + unc-119(+)]* | | | This study | |
| Lib2257 | *L210I* | *fat-1(wa9); kunEx327[Pvha-6::fat-1(L210I)::6× His-tag::gfp + unc-119(+)]* | | | This study | |
| Lib2554 | *F211V* | *fat-1(wa9); kunEx328[Pvha-6::fat-1(F211V)::6× His-tag::gfp +unc-119(+)]* | | | This study | |
| Lib2556 | *G212M* | *fat-1(wa9); kunEx329[Pvha-6::fat-1(G212M)::6× His-tag::gfp + unc-119(+)]* | | | This study | |
| Lib2562 | *F213L* | *fat-1(wa9); kunEx330[Pvha-6::fat-1(F213L)::6× His-tag::gfp + unc-119(+)]* | | | This study | |
| Lib2566 | *C214G* | *fat-1(wa9); kunEx331[Pvha-6::fat-1(C214G)::6× His-tag::gfp + unc-119(+)]* | | | This study | |
| Lib2569 | *D215M* | *fat-1(wa9); kunEx332[Pvha-6::fat-1(D215M)::6× His-tag::gfp + unc-119(+)]* | | | This study | |
| Lib2572 | *G216L* | *fat-1(wa9); kunEx333[Pvha-6::fat-1(G216L)::6× His-tag::gfp + unc-119(+)]* | | | This study | |
| Lib2466 | *S217R* | *fat-1(wa9); kunEx334[Pvha-6::fat-1(S217R)::6× His-tag::gfp + unc-119(+)]* | | | This study | |
| Lib2270 | *S230Q* | *fat-1(wa9); kunEx335[Pvha-6::fat-1(S230Q)::6× His-tag::gfp + unc-119(+)]* | | | This study | |
| Lib2396 | *L378M-D379N* | *fat-1(wa9);kunEx336[Pvha-6::fat-1(L378M-D379N)::6× His-tag::gfp + unc-119(+)]* | | | This study | |
| Lib3087 | *H168V-K171H* | *fat-1(wa9);kunEx337[Pvha-6::fat-1(H168V-K171H)::6× His-tag::gfp + unc-119(+)]* | | | This study | |
| Lib3086 | *W264Y* | *fat-1(wa9); kunEx338[Pvha-6::fat-1(W264Y)::6× His-tag::gfp + unc-119(+)]* | | | This study | |
| Lib3085 | *K190E* | *fat-1(wa9); kunEx339[Pvha-6::fat-1(K190E)::6× His-tag::gfp + unc-119(+)]* | | | This study | |
| **Cells** | | |  |  | |  |
| **Name** | | | **Short name in this study** | **Source** | |  |
| HEK-293T | | |  | ATCC | |  |
| pCDH | | | EV | This study | |  |
| pCDH-FAT-1-FLAG | | | FAT-1 | This study | |  |
| pCDH-FAT-1(*G212M*)-FLAG | | | *G212M* | This study | |  |
| pCDH-FAT-1(*G216L*)-FLAG | | | *G216L* | This study | |  |
| pCDH-FAT-1(*S217R*)-FLAG | | | *S217R* | This study | |  |

Notes: CGC indicates Caenorhabditis Genetics Center.

**Supplemental Table S2 DNA primers used in this study.**

| **Primer name** | **Sequence** | **Note** |
| --- | --- | --- |
| ***Pfat-1::fat-1::6× his::gfp* constructs** | | |
| *pCFJ151+fat-1 F2* | ATCCACGAAGCTTCCCATGGCTCATCTCACTCAGGCATGC | For *Pfat-1::fat-1* fragment amplification |
| *Histag+fat-1 R* | ATGGTGATGGTGATGATGCTTGGCCTTTGCCTTCTCC |  |
| *Histag+GFP F1* | CATCATCACCATCACCATTTGCATGCCTGCAGGTCGAC | For *pCFJ151+6×* *his::gfp+GFP* fragment amplification |
| *pCFJ151 R1* | CCATGGGAAGCTTCGTGGAT |  |
| ***Pvha-6::fat-1::6× his::gfp* constructs** | | |
| *pCFJ151+vha-6 F* | ATCCACGAAGCTTCCCATGGGTAGAGCATGTACCTTTATAGG | For *vha-6* fragment amplification |
| *fat-1+vha-6 R* | CTGAGGAATGAGCGACCATGTAGGTTTTAGTCGCCCTG |  |
| *vha-6+fat-1 F* | CAGGGCGACTAAAACCTACATGGTCGCTCATTCCTCAG | For *fat-1* fragment amplification |
| *GFP+his+fat-1 R* | GTCGACCTGCAGGCATGCAAATGGTGATGGTGATGATGCTTGGCCTTTGCCTTCTCC |  |
| *Histag+GFP F1* | CATCATCACCATCACCATTTGCATGCCTGCAGGTCGAC | For *pCFJ151+6× his::gfp+GFP* fragment amplification |
| *pCFJ151 R1* | CCATGGGAAGCTTCGTGGAT |  |
| ***Pvha-6::fat-1(∆1-100 aa)::6× his::gfp* constructs** | | |
| *fat-1(∆1-100 aa) F* | TTGTTTGGTTACTTGGTTTGGAAC | For *fat-1(∆1-100)* fragment amplification |
| *fat-1(∆1-100 aa) R* | GTTCCAAACCAAGTAACCAAACAAGTAGGTTTTAGTCGCCCTG |  |
| ***Pvha-6::fat-1(∆103-351 aa)::6× his::gfp* constructs** | | |
| *fat-1(∆103-351 aa) F* | CTTTTGAGTACTTTGGATTGTTTTTGTCCGACACCCAATACGG | For *fat-1(∆103-351)* fragment amplification |
| *fat-1(∆103-351 aa) R* | GTACCCGTATTGGGTGTCGGACAAAAACAATCCAAAGTACTC |  |
| ***Pvha-6::fat-1(Y90T)::6× his::gfp* constructs** | | |
| *fat-1(Y90T) F* | TCTCACATTTGCTCTTCCAGC | For *fat-1(Y90T)* fragment amplification |
| *fat-1(Y90T) R* | GCAAATGTGAGAATTGTGAG |  |
| ***Pvha-6::fat-1(V176P-I178V)::6× his::gfp* constructs** | | |
| *fat-1(V176P-I178V) F* | GACACCCATGGGTTCAGGATAAGGATTGGG | For *fat-1(V176P-I178V)* fragment amplification |
| *fat-1(V176P-I178V) R* | CCTGAACCCATGGGTGTCCATGATCTTTGTC |  |
| ***Pvha-6::fat-1(∆210-217 aa)::6× his::gfp* constructs** | | |
| *fat-1(∆210-217 aa) F* | CACTTCTGGCCATACTCTTCACTTTTTGTTCG | For *fat-1(∆210-217)* fragment amplification |
| *fat-1(∆210-217 aa) R* | GTGAAGAGTATGGCCAGAAGTGAGTGTACACTGGGAACCATTTAAG |  |
| ***Pvha-6::fat-1(L210I)::6× his::gfp* constructs** | | |
| *fat-1(L210I) F* | CACTATCTTCGGTTTCTGTG | For *fat-1(L210I)* fragment amplification |
| *fat-1(L210I) R* | CGAAGATAGTGTACACTGGG |  |
| ***Pvha-6::fat-1(F211V)::6× his::gfp* constructs** | | |
| *fat-1(F211V) F* | CTTTAGTCGGTTTCTGTGATGGATCTCAC | For *fat-1(F211V)* fragment amplification |
| *fat-1(F211V) R* | AACCGACTAAAGTGTACACTGGGAACC |  |
| ***Pvha-6::fat-1(G212M)::6× his::gfp* constructs** | | |
| *fat-1(G212M) F* | ATTCATGTTCTGTGATGGATCTCACTTCTG | For *fat-1(G212M)* fragment amplification |
| *fat-1(G212M) R* | AGAACATGAATAAAGTGTACACTGGGAACC |  |
| ***Pvha-6::fat-1(F213L)::6× his::gfp* constructs** | | |
| *fat-1(F213L) F* | CGGTCTTTGTGATGGATCTCACTTCTGG | For *fat-1(F213L)* fragment amplification |
| *fat-1(F213L) R* | CACAAAGACCGAATAAAGTGTACACTGGG |  |
| ***Pvha-6::fat-1(C214G)::6× his::gfp* constructs** | | |
| *fat-1(C214G) F* | TTTCGGTGATGGATCTCACTTCTGGCC | For *fat-1(C214G)* fragment amplification |
| *fat-1(C214G) R* | CATCACCGAAACCGAATAAAGTGTACAC |  |
| ***Pvha-6::fat-1(D215M)::6× his::gfp* constructs** | | |
| *fat-1(D215M) F* | CTGTATGGGATCTCACTTCTGGCCATAC | For *fat-1(D215M)* fragment amplification |
| *fat-1(D215M) R* | ATCCCATACAGAAACCGAATAAAGTGTAC |  |
| ***Pvha-6::fat-1(G216L)::6× his::gfp* constructs** | | |
| *fat-1(G216L) F* | TGATCTCTCTCACTTCTGGCCATACTC | For *fat-1(G216L)* fragment amplification |
| *fat-1(G216L) R* | GAGAGAGATCACAGAAACCGAATAAAG |  |
| ***Pvha-6::fat-1(S217R)::6× his::gfp* constructs** | | |
| *fat-1(S217R) F* | TGGACGTCACTTCTGGCCATAC | For *fat-1(S217R)* fragment amplification |
| *fat-1(S217R) R* | GAAGTGACGTCCATCACAGAAACCG |  |
| ***Pvha-6::fat-1(S230Q)::6× his::gfp* constructs** | | |
| *fat-1(S230Q) F* | GTAACCAGGAACGTGTTCAATG | For *fat-1(S230Q)* fragment amplification |
| *fat-1(S230Q) R* | GTTCCTGGTTACGAACAAAAAG |  |
| ***Pvha-6::fat-1(L378M-D379N)::6× his::gfp* constructs** | | |
| *fat-1(L378M-D379N) F* | CAAGATGAATTATCTCGTTCACAAGACCG | For *fat-1(L378M-D379N)* fragment amplification |
| *fat-1(L378M-D379N) R* | GATAATTCATCTTGTAGTTGAACCACAGG |  |
| ***Pvha-6::fat-1(H168V-K171H)::6× his::gfp* constructs** | | |
| *fat-1(H168V-K171H) F* | CCAACGTAATTGACCATGATCATGGACACGTGTGGATTC | For *fat-1(H168V-K171H)* fragment amplification |
| *fat-1(H168V-K171H) R* | CACGTGTCCATGATCATGGTCAATTACGTTGGTG |  |
| ***Pvha-6::fat-1(W264Y)::6× his::gfp* constructs** | | |
| *fat-1(W264Y) F* | CTATTACGTTCCACTTTCTTTCTTCGG | For *fat-1(W264Y)* fragment amplification |
| *fat-1(W264Y) R* | GGAACGTAATAGTACCAGAACCAATTGG |  |
| ***Pvha-6::fat-1(K190E)::6× his::gfp* constructs** | | |
| *fat-1(K190E) F* | CATGGGAGAGATGGTTCAATCCAATTCC | For *fat-1(K190E)* fragment amplification |
| *fat-1(K190E) R* | GGATTGAACCATCTCTCCCATGATGGCATTGCTTCC |  |
| ***pCDH-FAT-1-FLAG* constructs** | | |
| *pCDH-fat-1 C.elegans F* | ATGACGATGACAAGTCTAGAGAATTCATGGTCGCTCATTCCTCAGAAG | For *FAT-1* fragment amplification |
| *pCDH-fat-1 C.elegans R* | ATCGCAGATCCTTCGCGGCCGCCTTGGCCTTTGCCTTCTCCTCG |  |
| ***pCDH-FAT-1(G212M)-FLAG* constructs** | | |
| *fat-1(G212M) F* | ATTCATGTTCTGTGATGGATCTCACTTCTG | For *FAT-1(G212M)* fragment amplification |
| *fat-1(G212M) R* | AGAACATGAATAAAGTGTACACTGGGAACC |  |
| ***pCDH-FAT-1(G216L)-FLAG* constructs** | | |
| *fat-1(G216L) F* | TGATCTCTCTCACTTCTGGCCATACTC | For *FAT-1(G216L)* fragment amplification |
| *fat-1(G216L) R* | GAGAGAGATCACAGAAACCGAATAAAG |  |
| ***pCDH-FAT-1(S217R)-FLAG* constructs** | | |
| *fat-1(S217R) F* | TGGACGTCACTTCTGGCCATAC | For *FAT-1(S217R)* fragment amplification |
| *fat-1(S217R) R* | GAAGTGACGTCCATCACAGAAACCG |  |

**Supplemental Table S3 Regents used in this study.**

| **Name** | **Product number** | **Source** |
| --- | --- | --- |
| **Antibodies** | | |
| Anti-FLAG (Mouse) | F3165 | Sigma-Aldrich |
| Anti-GFP (Rabbit) | HY-P80141 | MCE |
| Anti-GAPDH (Mouse) | 60004-1-IG | Proteintech |
| Anti-β-actin (Mouse) | 66009-1-IG | Proteintech |
| **Fatty acids** | | |
| C18:2n6 | CDDE-U-59-A-1GM | ANPEL |
| C18:3n6 | CDAA-253189A | ANPEL |
| C20:3n6 | CDAA-253205M | ANPEL |
| C20:4n6 | CDDE-U-71-A-1GM | ANPEL |
